# They Don't Even Know Your Name: An Intergenerational Modeling Deficit in Patient-Centered Practice

**DOI:** 10.7759/cureus.113909

**Published:** 2026-08-03

**Authors:** Lourdes V Quiles-Sánchez, Georgios Kyriakos, Juan F Menárguez-Puche, Juan A Sánchez-Sánchez, Miriam Moñino-García

**Affiliations:** 1 Roldán Primary Care Health Centre, Servicio Murciano de Salud, Murcia, ESP; 2 Department of Endocrinology and Nutrition, Hospital General Universitario Santa Lucía, Cartagena, ESP; 3 Molina-Jesús Marín Primary Health Centre, Servicio Murciano de Salud, Murcia, ESP; 4 Department of Social and Health Sciences, Faculty of Medicine, Universidad de Murcia, Murcia, ESP; 5 Department of Public Health Sciences, Faculty of Medicine, Universidad de Murcia, Murcia, ESP

**Keywords:** hidden curriculum, intergenerational modeling deficit, medical education, patient-centered care, qualitative research

## Abstract

Introduction

This study explored how hidden curriculum mechanisms shape patient-centered care (PCC) attitudes among medical students in a Spanish public medical school with minimal formal PCC instruction, and how these mechanisms may be perceived to reproduce themselves when faculty PCC development is limited.

Methods

We conducted 11 focus groups with 93 medical students (83 third-year preclinical and 10 sixth-year clinical students; the sixth-year group was treated as exploratory). Sessions were audio- and video-recorded, transcribed verbatim, and analyzed using qualitative content analysis. Reporting followed established qualitative research guidelines.

Results

Five categories were identified: (1) formal curriculum gaps despite sophisticated PCC understanding; (2) disease-centered and paternalistic teaching orientation linked to perceived defensive practice norms; (3) role modeling functioning as the dominant PCC pedagogy in the absence of structured training; (4) tension between PCC ideals and clinical adaptation, including anticipatory erosion narratives among preclinical students; and (5) protective factors (intrinsic motivation, family health experiences, and critical appraisal of the training environment) that sustained PCC commitments. Participants described what we term an intergenerational modeling deficit: faculty perceived as lacking PCC training were viewed as unable to model patient-centered practice, contributing to a self-reinforcing cycle across training generations.

Conclusions

The intergenerational modeling deficit is offered as a heuristic proposition that may help explain the persistence of hidden curriculum effects and highlights faculty development focused on observable modeling behaviors as a potential intervention target.

## Introduction

Patient-centered care (PCC) is a foundational principle of contemporary medicine. Mead and Bower’s framework defines PCC through five dimensions - the biopsychosocial perspective, the patient-as-person, sharing power, the therapeutic alliance, and the doctor-as-person [1] - and international competency frameworks have enshrined it as a core educational outcome [2]. Empathic consultations improve clinical outcomes, treatment adherence, patient satisfaction, and physician well-being while reducing costs [3].

Yet medical students’ patient-centered attitudes and empathy decline as training progresses [4,5]. Hafferty identified the hidden curriculum - the institutional culture, informal socialization, and tacit norms - as a powerful counterforce to formal teaching [6-8], operating through role models, hierarchies, time pressures, and assessment culture [6,7]. These mechanisms map onto specific PCC dimensions: throughput culture and time pressure weaken the patient-as-person dimension and therapeutic alliance; hierarchical paternalism constrains the sharing of power; and disease-centered socialization marginalizes the biopsychosocial perspective. Understanding this mapping is essential for designing targeted interventions.

Recent evidence has sharpened understanding. Howick and colleagues’ systematic review identified overwork, cynical role models, and lack of support as the primary empathy-decline drivers [9]. The same group proposed an empathic hidden curriculum roadmap [10], reported empathy decline at the preclinical-to-clinical transition [11], and showed that a transition course can reverse it [12]. A scoping review of 29 studies confirmed role modeling and institutional culture as central pathways [13]. Complementary research demonstrated the role of emotional socialization [14], professionalism dilemmas as hidden curriculum tensions [15], role modeling as the widely documented transmission pathway [16], PCC decline during clerkships even in communication-trained students [17], and empathy training effectiveness [18].

However, this evidence is geographically concentrated in Anglophone settings [9]. A recent scoping review of 29 studies across 10 countries included no studies from Southern Europe [13]. In Spain, medical schools dedicate only 2.77 European Credit Transfer System (ECTS) to communication skills versus the 5.0 recommended [19], and academic leaders identify pervasive barriers including time constraints, lack of faculty training, and institutional resistance [20]. The Spanish public healthcare system’s structural features - high patient volumes, brief consultation times, and medico-legal pressures - may create a particularly challenging environment for PCC socialization. The Spanish Patient-Practitioner Orientation Scale (PPOS) has been validated [21], and a meta-analysis confirmed suboptimal PCC attitudes globally [22], but quantitative approaches cannot illuminate the formation mechanisms through which the hidden curriculum operates in specific training contexts.

This study has explored how hidden curriculum mechanisms shape PCC attitudes among medical students at a Spanish public university where formal PCC training is minimal (~3/360 ECTS credits), creating conditions under which informal socialization dominates. We were particularly interested in how hidden curriculum effects may reproduce themselves when faculty development in PCC is largely absent. A search of PubMed, Scopus, and Dialnet identified no prior Spanish qualitative studies examining hidden curriculum mechanisms shaping PCC attitudes in medical students.

## Materials and methods

Study design

**Figure 1:**
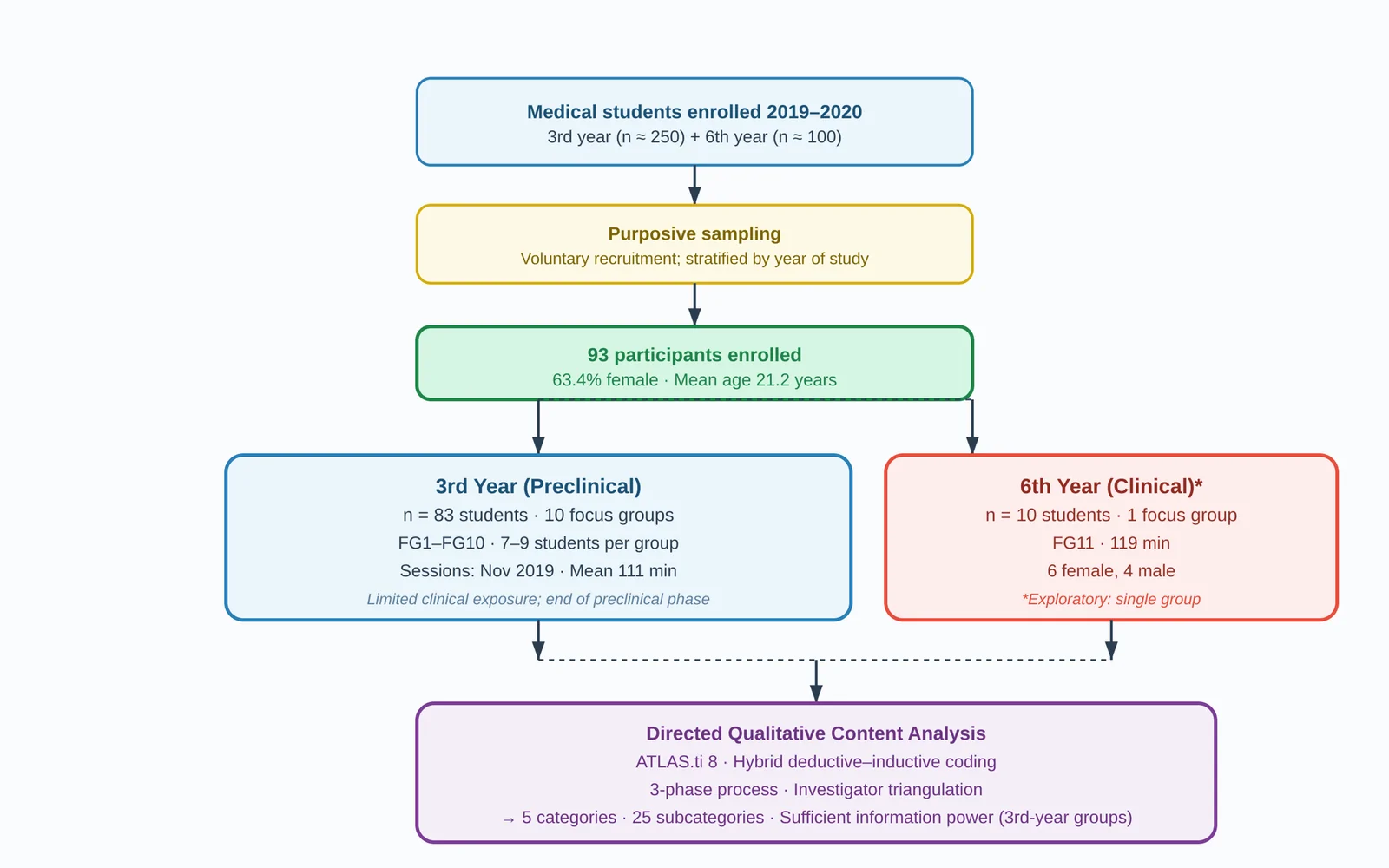
Participant recruitment, focus group allocation, and analytic flow Ninety-three medical students participated across 11 focus groups: 10 third-year groups (n=83, preclinical) and one sixth-year group (n =10, clinical). *Sixth-year data are exploratory (single focus group). ECTS: European Credit Transfer System; PCC: patient-centered care. Figure created using Python (Python Software Foundation, Wilmington, DE) matplotlib (v3.8, The Matplotlib Development Team).

This qualitative study used focus groups and directed qualitative content analysis [23] with a hybrid deductive-inductive coding strategy [24]. Initial coding was informed by sensitizing concepts (hidden curriculum, PCC dimensions) and topic-guide domains; subcategories were developed inductively through iterative comparison across transcripts. Focus groups were chosen because group dynamics enable shared norms to surface naturally [25]. Reporting follows the Consolidated Criteria for Reporting Qualitative Research (COREQ) [26]; the completed checklist is available from the corresponding author upon request. Figure 1 presents the recruitment and analytic flow.

Setting and context

The study was conducted at the Faculty of Medicine, Universidad de Murcia - a six-year program with three preclinical and three clinical years. PCC content is limited to Psychology (six ECTS), Bioethics (three ECTS), General Pathology, and Family Medicine; no standalone PCC module exists. This curricular structure has remained unchanged from 2019 through 2025, although the clinical learning environment may have been affected by factors including the COVID-19 pandemic. The dataset thus captures a pre-COVID baseline of hidden curriculum mechanisms in one Spanish medical faculty.

Participants and sampling

Ninety-three students participated across 11 focus groups: 83 third-year (preclinical) and 10 sixth-year students (clinical; single focus group was treated as exploratory). The sample was 63.4% women with a mean age of 21.2 years (Table 1).

**Table 1:** Characteristics of the 11 focus groups (n = 93) FG: focus group. All sessions conducted in November 2019 at the Faculty of Medicine, Universidad de Murcia. Third-year students were in their final preclinical year; the sixth-year group (FG11) was treated as exploratory given its single-group design. Duration reported in minutes.

Group	Year	n	Female	Male	Duration (min)	Phase
FG1	3rd	8	7	1	115	Preclinical
FG2	3rd	9	5	4	100	Preclinical
FG3	3rd	8	5	3	124	Preclinical
FG4	3rd	8	5	3	107	Preclinical
FG5	3rd	7	3	4	124	Preclinical
FG6	3rd	9	6	3	124	Preclinical
FG7	3rd	9	5	4	91	Preclinical
FG8	3rd	9	8	1	110	Preclinical
FG9	3rd	8	5	3	90	Preclinical
FG10	3rd	8	4	4	120	Preclinical
FG11	6th	10	6	4	119	Clinical
Total		93	59	34	111 (mean)	

Data collection

Eleven sessions were conducted in November 2019 by LVQS and MMG as the principal investigators, assisted by trained postgraduate health sciences students, who had completed training in focus group moderation. All sessions were conducted in Spanish at the Faculty of Medicine. A semi-structured topic guide (available from the corresponding author upon request) covered five domains: PCC knowledge, communication skills, attitudes and teaching orientation, motivation and expectations, and future applicability. The mean duration of the sessions was 111 minutes (range: 90-124 minutes). Groups comprised six to 12 participants, consistent with recommended focus group size [25]. All sessions were audio- and video-recorded with written consent. Third-year groups were conducted separately from the sixth-year group to minimize power dynamics between training stages.

Data analysis

Transcripts were analyzed using ATLAS.ti 8 (ATLAS.ti Scientific Software Development GmbH, Berlin) through three phases: deductive coding from topic-guide domains, inductive emergence of new subcategories, and recursive refinement. Two analysts independently coded a subset of transcripts; disagreements were resolved through discussion until consensus was achieved. Across the 10 third-year groups, later discussions yielded no substantively new categories, suggesting sufficient information power for the study aims. The single sixth-year group was analyzed as exploratory comparative material. Codebook available from the corresponding author upon request.

Researcher positionality

The research team comprised a physician and doctoral candidate (LVQS), who moderated all focus group sessions together with MMG and trained postgraduate health sciences students, and two senior supervisors with expertise in medical education and qualitative methods, respectively. LVQS had no teaching or examining role with participants, although her clinical background was disclosed to students. The team recognized an a priori expectation that PCC attitudes would erode during clinical training and documented this assumption in analytic memos. Debriefing meetings after every third transcript focused explicitly on disconfirming cases - for example, accounts of positive role modeling and sustained patient-centered commitments - which informed codebook refinement and prevented premature closure around an erosion-only narrative.

Trustworthiness

Credibility was supported through analyst triangulation (independent coding of initial transcripts, followed by joint refinement) and prolonged engagement with the dataset. Transferability was supported through a thick description of the setting and participants, enabling readers to assess applicability to other contexts. Dependability was ensured through a systematic audit trail in ATLAS.ti, documenting all the coding decisions. Confirmability was strengthened through audit trail documentation, analyst discussion, and consideration of a parallel PPOS survey as contextual corroboration - rather than a direct confirmation - of qualitative themes. Member checking was not conducted; to compensate, the team used negative case analysis and iterative peer debriefing.

Ethics statement

This study was approved by the Ethics Committee of the Faculty of Medicine, Universidad de Murcia. Written informed consent was obtained from the participants. Participation was voluntary.

## Results

Five categories were identified, with recurring links across participants' accounts (Table 2). Below, we trace a narrative arc from curricular gaps through hidden curriculum exposure to adaptation and resistance, highlighting the process and interconnection rather than isolated themes. Quotations were translated from Spanish by LVQS, reviewed by a bilingual co-author, and checked against original transcripts to preserve semantic and pragmatic meaning; original Spanish quotations are available from the corresponding author upon request.

**Table 2:** Categories, subcategories, and illustrative quotations Five categories emerged from directed qualitative content analysis of 11 focus groups (n=93). Subcategories listed in order of prominence within each category. Quotations translated from Spanish (original Spanish quotations are available upon request). FG: focus group; P: participant; PCC: patient-centered care.

Category	Subcategories	Illustrative quotation
1. Curriculum gaps	Limited subjects; Theoretical overload; No workshops; Preclinical only	“360 credits…three or four cover this.” (FG11-P1)
2. Teaching orientation	Disease-centered; Paternalistic; Defensive medicine; Intergenerational transmission	“Paternalistic and increasingly informative.” (FG11-P3)
3. Role modeling	Negative (vivid); Positive (rare, transformative); Teacher variability; Specialty modulation	“If your tutor teaches you this…you’ll end up doing that.” (FG11-P2)
4. Progressive erosion	Time pressure; Technology; Hierarchy; Assessment culture; Specialty avoidance	“The system forces you to overlook the personal aspect.” (FG2-P2)
5. Protective factors	Intrinsic motivation; Family health; Peer support; PCC benefits; Balanced practice	“Being something more than someone who just prescribes.” (FG5-P3)

"We understand the idea, but where is it taught?"

Students demonstrated a sophisticated understanding of PCC, yet described a striking disjunction between conceptual literacy and curricular visibility. This gap sets the stage for the hidden curriculum's influence: when formal teaching is marginal, clinical observation becomes the dominant pedagogy:

"The curriculum has 360 credits. In how many subjects is this covered? In Ethics, which has three, and in Psychology, which has six… So what does it come down to-three or four credits out of 360? And then we're going to treat people" (FG11-P1, sixth year, female student).

"This degree has an extraordinary theoretical overload, and in many cases unnecessary - there are things that with 10 minutes of practice in a hospital you learn for life" (FG5-P5, third year, male student).

These accounts were echoed across multiple third-year groups, revealing a shared awareness that formal PCC teaching occupied a marginal curricular position. Students could articulate PCC principles with considerable sophistication - consistent with Mead and Bower's dimensions - yet perceived no structured opportunity to practice or receive feedback on these skills. This disjunction is analytically significant: when formal teaching provides conceptual knowledge but no enacted practice, students possess the vocabulary to recognize patient-centeredness but lack the pedagogical scaffolding to develop it as a clinical competence. The vacuum this creates is not neutral - it is filled by whatever modeling is available in the clinical environment, making the hidden curriculum the de facto PCC pedagogy. In other groups, students expressed this gap more directly: "We really lack training when it comes to handling certain special situations" (FG10-P5, third year, male student).

"Not because the subject matter connects to that relationship, but because you actually find yourself in a real situation where you have to treat, talk to, or ask a real person" (FG4-P5, third year, female student).

Encountering the hidden curriculum: role models and teaching orientation

Participants described disease-centered, paternalistic teaching and linked this to what they perceived as a defensive medicine orientation in their training context:

"I think the attitude is paternalistic and increasingly informative - like 'I'm covering myself, I'm playing it safe'" (FG11-P3, sixth year, male student).

Role modeling emerged as the dominant pathway through which the hidden curriculum operated, consistent with international evidence [16], but functioning here as virtually the only PCC pedagogy in the absence of formal training. This distinction matters: in settings where communication skills courses exist alongside clinical placements, role modeling competes with formal instruction. In the context studied, where PCC occupies less than 1% of the curriculum, role modeling has no formal counterweight. What students observe in clinical encounters becomes what they learn about patient-centered practice - for better or worse:

"I think the most important thing is what you see in clinical practice. If your tutor teaches you this over a period of time, you'll end up doing that" (FG11-P2, sixth year, female student).

The title quotation captures negative modeling (original Spanish: "la mayoría ni se saben tu nombre" (most of them don't even know your name)):

"My experience with both GPs and specialists is that most of them don't even know your name, and they don't show much interest in knowing how something affects your life" (FG5-P4, third year, female student).

Several participants described what appeared to be an intergenerational modeling deficit - teachers who themselves lacked PCC training could not model what they had never learned:

"They learned by trial and error. Nobody taught them even the minimum of what they're teaching us" (FG11-P1, sixth year, female student).

The intergenerational pattern was described in both preclinical and the exploratory clinical group, though with different registers. Third-year students framed it as an anticipated problem ("what will happen when we become doctors?"), whereas the sixth-year participant quoted above spoke from a direct observation of teachers' limitations. Taken together, these accounts point to a self-reinforcing mechanism: faculty who were never trained in PCC cannot model it; students, lacking formal alternatives, adapt to what they observe; those students eventually become faculty who perpetuate the same absence. Unlike a simple training gap - which could be resolved by training one cohort of faculty - this mechanism predicts that the deficit will reproduce itself across generations unless the cycle is interrupted at the level of observable modeling behavior. This extends beyond individual role-modeling effects to implicate a systemic failure in faculty preparation.

The process of erosion

Across groups, students described tension between patient-centered ideals and clinical adaptation. A notable finding among preclinical students was the articulation of erosion narratives before full clinical immersion, suggesting anticipatory socialization through near-peer accounts or early observational placements. The exploratory sixth-year group described this process in more concrete, experiential terms:

"The mechanization of the profession - when a patient arrives and you don't even look at them because you're typing on the computer, and you treat it like 'I touch this or prescribe that and it seems like I've solved it.' But active listening is very important" (FG8-P3, third year, female student).

"Once you get more involved in the hospital environment, the system forces you in many ways to overlook the personal aspect - not because you forget or don't want to, but because there simply isn't time" (FG2-P2, third year, male student).

"I know classmates who are going into specialties with no patient contact, and they tell you openly: 'I prefer this because I prefer not to see their face'" (FG11-P8, sixth year, male student).

The first erosion quotation comes from a third-year student - someone with limited clinical exposure who nonetheless articulated system-driven erosion in vivid terms. This anticipatory socialization is analytically significant as a standalone finding: hidden curriculum influence appears to begin before clinical immersion, transmitted through peer narratives, early observations, and cultural expectations about clinical life. The specialty-avoidance account from the exploratory sixth-year group illustrates a more extreme adaptation, where erosion progresses from attitudinal shift to career-shaping decisions - though this observation requires cautious interpretation given the single-group context.

Sustaining patient-centeredness

Despite erosion pressures, participants identified protective factors - including intrinsic vocational motivation and personal or family health experiences - that sustained patient-centered commitments. These accounts complicate a simple decline narrative and suggest potential intervention leverage points:

"Being something more than someone who just prescribes a treatment" (FG5-P3, third year, female student).

"You shouldn't create a separate subject for this. What's really important is educating people to have good clinical values" (FG11-P8, sixth year, male student).

The second quotation is analytically significant: a sixth-year student who has witnessed clinical erosion argues against creating a separate PCC subject, advocating instead for values integration throughout training. This represents a sophisticated negotiation of competing pressures - acknowledging the hidden curriculum's power while proposing structural rather than additive solutions. Such accounts suggest that protective factors operate not merely as individual resilience but as critical appraisal of the training environment, offering potential leverage for curricular reform from within the student body.

Cross-group patterns

Categories were present across the preclinical groups, with the exploratory sixth-year group providing contextual illustration rather than comparative data. Third-year participants discussed formal curriculum gaps and protective factors most extensively, often framing PCC as an aspiration and curricular absence as a missed opportunity. The intergenerational modeling deficit and defensive practice orientation appeared in both preclinical and clinical accounts but with different registers: preclinical students spoke in anticipatory terms, whereas the sixth-year participants drew on direct observation. These patterns should be read as illustrative rather than as evidence of stage-based trajectories, given the single sixth-year focus group.

## Discussion

Principal findings

This study examined how the hidden curriculum shapes PCC attitudes in a training context where formal PCC instruction occupies less than 1% of the curriculum, creating conditions under which informal socialization dominates. The central finding is what we term an intergenerational modeling deficit: faculty who themselves received little or no PCC training were perceived by students as unable to model patient-centered practice, producing a self-reinforcing cycle in which training deficits reproduce themselves across faculty generations. This mechanism was embedded within a broader pattern of hidden curriculum effects - role modeling as the dominant socialization pathway, disease-centered teaching orientations, anticipatory erosion among preclinical students, and protective factors that sustained patient-centered commitments. Table 3 positions these findings in relation to the international evidence.

**Table 3:** Positioning of present findings within the international evidence This table maps the study's five thematic areas against published international evidence to highlight convergences and novel contributions. Reference numbers in square brackets correspond to the reference list. HC: hidden curriculum; PCC: patient-centered care; ECTS: European Credit Transfer System. *Sixth-year findings are exploratory (single focus group).

Theme	International evidence	Participants' accounts (present study)	Relationship to literature
HC mechanisms driving PCC/empathy decline	Overwork, cynical role models, lack of institutional support [9,13]	Role modeling, time pressure, and hierarchy prominently discussed; defensive practice orientation additionally described	Core mechanisms resonate with international findings; defensive practice orientation was less emphasized in the reviews cited
Role modeling	Widely documented transmission pathway [16]	Most prominently discussed mechanism; participants described an intergenerational modeling deficit	The modeling deficit proposition was not identified in the reviews cited; it warrants prospective investigation
PCC attitude trajectory	Empathy decline during clinical training [5,11]	Preclinical students described idealism alongside anticipatory erosion narratives; exploratory sixth-year group described lived erosion*	Broad pattern resonates with international reports; stage-specific comparisons are exploratory in this dataset
Teaching orientation	Disease-centered orientation; formal-informal curriculum gap [9,13]	Paternalistic and disease-centered orientation described; formal PCC teaching occupies ~3/360 ECTS	Participants perceived a pronounced formal-informal gap, possibly reflecting the low ECTS allocation documented in this context [19]
Protective factors	Near-peer mentoring, empathy champions, transition courses [10,12]	Intrinsic motivation, family health experiences, critical appraisal of training culture	Student-identified factors suggest potential leverage points that parallel themes in the intervention literature
Systemic/structural barriers	Clinical environment characterized as hostile to empathy [9,17]; faculty barriers documented in Spain [20]	Participants described time pressure and institutional constraints as normalizing disease-centered practice	Perceptions echo the structural barriers documented internationally [9,17] and in Spanish institutional surveys [19,20]

What this study adds

Our findings contribute to the evidence base in three ways. First, and most centrally, participants described a dynamic we term an intergenerational modeling deficit: faculty who themselves received little or no PCC training were perceived as unable to model patient-centered practice, leading students to adapt to what they observed rather than what the formal curriculum espoused. This is more than a training gap - it is a self-reinforcing mechanism. Unlike a one-time deficit (which could be resolved by training a single cohort of faculty), the modeling deficit predicts that graduates socialized without PCC modeling will become faculty who perpetuate the same absence, reproducing the cycle across generations. We use "modeling deficit" as a shorthand for participants’ accounts of perceived capability gaps in their teachers, not as a measured faculty attribute. We offer this as a heuristic proposition that extends the existing socialization theory [27] by specifying a mechanism through which training deficits at the faculty level may reproduce themselves via daily modeling interactions. It merits prospective testing rather than treatment as a demonstrated causal mechanism. The pattern was described in preclinical groups and the exploratory clinical group, suggesting it may reflect a shared perception of how clinical teaching culture is transmitted, though participant accounts cannot establish the mechanism’s actual operation, and the single sixth-year group limits this inference.

Second, the study offers a contextual transferability assessment of hidden curriculum mechanisms in a training environment where formal PCC teaching is minimal, creating what may be understood as a "context of minimal formal instruction" in informal socialization. None of the 16 studies in Howick et al.’s qualitative synthesis [9] originated in Southern Europe, and a recent scoping review confirmed the geographic imbalance [13]. A Spanish qualitative study documented sustained humanistic vocation [28] but focused on meaning-making rather than analyzing hidden curriculum mechanisms directly. The mechanisms described by the participants - role modeling, hierarchy, and time pressure - also identified in Anglophone settings, are consistent with the possibility that core processes operate across training contexts, though further replication is needed. Third, students perceived features of their local training environment - including consultation time constraints and what they characterized as defensive practice norms - as intensifying hidden curriculum effects. These perceptions align with Ruiz Moral et al.’s survey of academic leaders across 30 Spanish medical schools, who identified structural and cultural barriers to communication skills teaching [20]. While our data cannot independently verify structural claims, the consistency between student perceptions and institutional-level evidence strengthens the case for context-sensitive hidden curriculum research.

The modeling deficit concept gains theoretical traction when situated within professional identity formation (PIF) scholarship [29,30]. Cruess et al.’s socialization model [27] positions role modeling as the primary vehicle through which professional identity is formed: students identify with clinical teachers and internalize their professional values and behaviors. Our data suggest that when PCC is systemically absent from available role models - not because individual faculty choose to devalue it, but because they were never equipped to enact it - the identity formation process may proceed without incorporating patient-centered practice as a professional norm. The modeling deficit thus operates at the intersection of structural training gaps and individual socialization processes: it is structural in origin (curricular absence of PCC faculty development) but individual in transmission (daily modeling interactions). This distinguishes it from Hafferty’s broader structural hidden curriculum [6] by specifying the mechanism through which structural effects are transmitted and reproduced at the individual level.

Bombeke et al. [17] documented PCC decline even among communication-trained Belgian students, suggesting that formal training alone may not protect against hidden curriculum effects when the modeling environment remains unchanged. The emotional dimension of clinical socialization described by Helmich et al. [14] helps contextualize why participants’ accounts carried affective weight: erosion was experienced not merely as cognitive adaptation but as a perceived loss of professional ideals - a finding consistent with PIF literature showing that identity threats provoke emotional responses that shape subsequent professional development.

A heuristic framework

**Figure 2:**
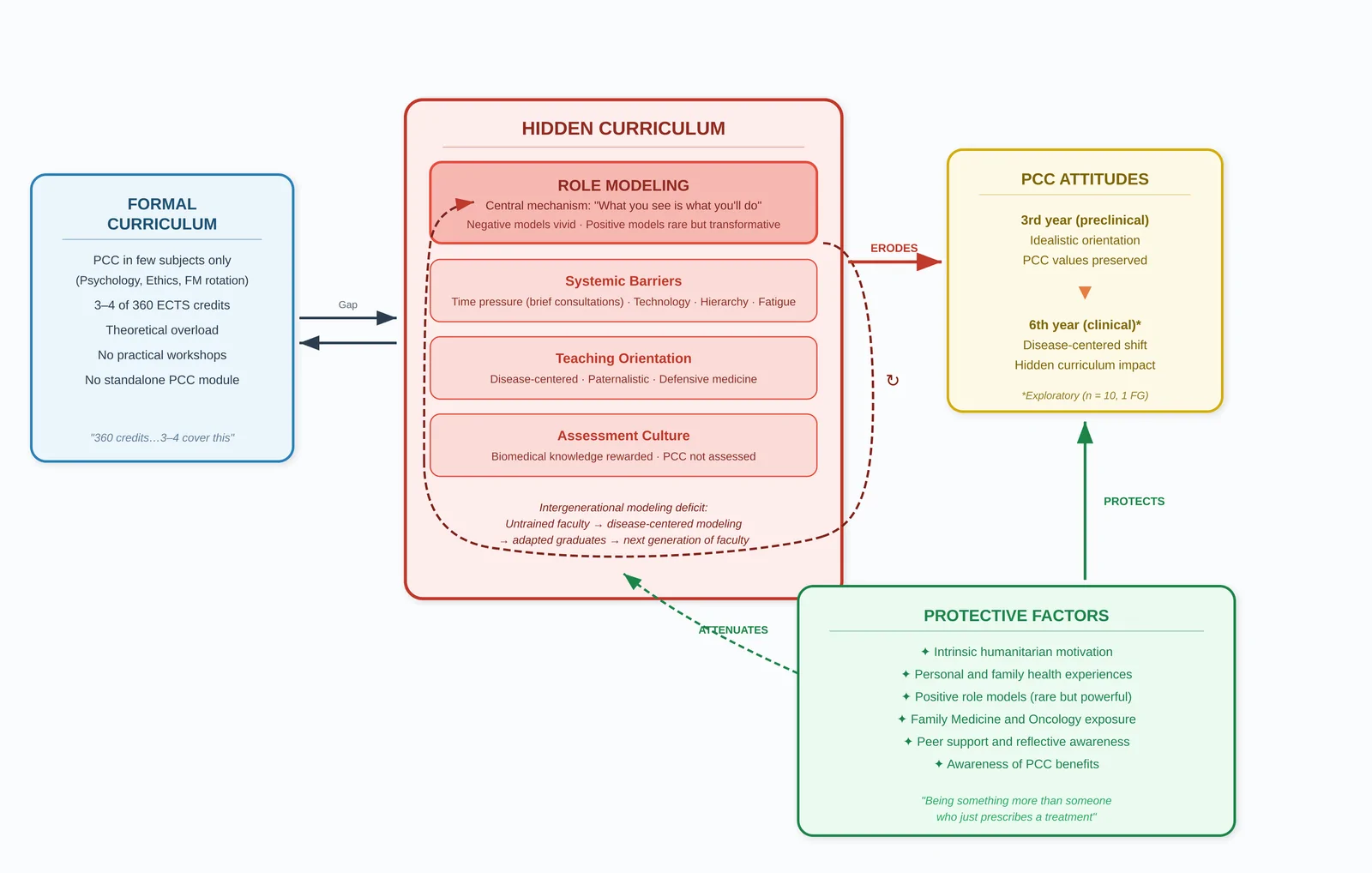
Heuristic framework of hidden curriculum mechanisms shaping patient-centered care attitudes The framework synthesizes participants' accounts and prior theory to illustrate how hidden curriculum processes may mediate between the formal curriculum and patient-centered care (PCC) attitudes, with the intergenerational modeling deficit positioned as the central self-reinforcing mechanism (dashed cycle). The mapping of specific hidden curriculum mechanisms onto Mead and Bower's PCC dimensions represents an author-imposed hypothesis requiring empirical testing. Intended as a practical heuristic to inform faculty development and curricular design, not as a tested causal model. *Sixth-year findings are exploratory (single focus group). ECTS: European Credit Transfer System; FG: focus group; FM: family medicine. Figure created using Python matplotlib (v3.8, The Matplotlib Development Team).

Figure 2 presents a heuristic framework illustrating how hidden curriculum processes may mediate between the formal curriculum and PCC attitudes, with the intergenerational modeling deficit positioned as the central self-reinforcing mechanism. The framework is informed by participants’ accounts and prior theory [1,6]. While it incorporates Mead and Bower’s PCC dimensions [1] as a theoretical reference - suggesting, for example, that throughput culture may weaken the patient-as-person dimension and that hierarchical paternalism may constrain sharing of power - we note that this mapping was not part of participants’ accounts and represents an author-imposed hypothesis requiring empirical testing. We present the framework as a heuristic proposition intended to orient future inquiry rather than to represent tested relationships. Its components require examination in other contexts and through longitudinal designs capable of tracking the processes it depicts.

Implications for practice

Two priorities warrant consideration, with the caveat that implications drawn from a single-site exploratory study require adaptation to local contexts. First, if the modeling deficit is operative, faculty development interventions that assume faculty already know what PCC looks like will be insufficient because the deficit is one of capability, not willingness. Interventions must therefore provide explicit PCC behavioral models and create observation opportunities where faculty can see patient-centered practice enacted before they are expected to model it. This shifts the target from "training faculty to be better communicators" to "ensuring faculty have ever seen the modeling they are expected to provide." Participants’ accounts suggest that faculty may need to see and practice these behaviors in situ, supported by observation and feedback, rather than receiving decontextualized communication tips. Feasible starting points include training in observable patient-centered micro-practices - introducing oneself, using patient names, agenda setting, eliciting impact on daily life, and narrating clinical reasoning at the bedside. Howick et al. [12] report a targeted educational intervention designed to protect empathy during transition into clinical training. In parallel, scoping evidence maps the breadth of hidden curriculum processes and highlights domains amenable to institutional attention [8]. Priority targets would include clerkship tutors, residents in high-contact specialties, and clinical supervisors whose daily practice constitutes the hidden curriculum that students observe.

Second, longitudinal PCC embedding across the curriculum could replace the current model, which confines PCC to preclinical credits. If the modeling deficit reproduces itself because students never see PCC enacted by faculty, then embedding PCC into clinical rotations - where faculty-student modeling interactions occur - is essential to interrupting the cycle. Specific opportunities include transition-to-clerkship workshops, structured reflective debriefs during clinical rotations, and observed clinical teaching audits that serve both formative development and institutional diagnosis of hidden curriculum patterns.

Strengths and limitations

The strengths of this study include a multi-group dataset (93 participants across 11 focus groups), an explicit deductive-inductive analytic strategy, and multiple credibility strategies including analyst triangulation, negative case analysis, and peer debriefing. Several limitations warrant transparent acknowledgment. This is a single-institution study with data collected in 2019. While the curricular structure remains unchanged and the mechanisms identified are consistent with 2023-2025 international evidence [9,13], clinical socialization conditions may have shifted post-COVID through changes in clinical contact patterns, digital learning, and evolving student discourse around professional identity. We interpret ongoing relevance at the level of structural mechanisms (role modeling, hierarchy, time pressure) rather than prevalence or magnitude; our findings should not be read as a current-state description of this institution in 2026 but as evidence about processes whose persistence is supported by recent reviews. The third-year predominance (83 vs 10 participants) means that any stage-related observations are exploratory and non-generalizable. The single sixth-year focus group cannot support robust cross-stage inference, and within-group dynamics in that session may have shaped findings. Focus groups may amplify normative discourse and participants may have performed patient-centered ideals rather than revealing private attitudes. Claims regarding defensive practice orientation and perceived structural barriers reflect participant perceptions and contextual interpretation, not independently observed system characteristics. Finally, member checking was not conducted; negative case analysis and peer debriefing were used as compensatory credibility strategies.

## Conclusions

The hidden curriculum shapes PCC attitudes in this Spanish setting through mechanisms that resonate with international evidence, but the central finding is a proposed intergenerational modeling deficit: a self-reinforcing cycle in which faculty who lack PCC training cannot model patient-centered practice, producing graduates who perpetuate the same absence. This mechanism, if confirmed through prospective and multi-site research, may explain why hidden curriculum effects persist despite decades of awareness and may represent a specific and interruptible target for faculty development interventions focused on observable modeling behavior rather than attitudes alone.
